# Pre-operative simulation of periacetabular osteotomy via a three-dimensional model constructed from salt

**DOI:** 10.1051/sicotj/2016051

**Published:** 2017-02-13

**Authors:** Kensuke Fukushima, Naonobu Takahira, Katsufumi Uchiyama, Mitsutoshi Moriya, Masashi Takaso

**Affiliations:** 1 Department of Orthopedic Surgery, Kitasato University School of Medicine 1-15-1 Kitasato Minami-ku, Sagamihara, Kanagawa 252-0374 Japan; 2 Department of Rehabilitation, Kitasato University School of Allied Health Sciences 1-15-1 Kitasato Minami-ku, Sagamihara, Kanagawa 252-0374 Japan

**Keywords:** Pre-operative simulation, Prototype model, Periacetabular osteotomy, Developmental dysplasia of the hip, Femoroacetabular impingement

## Abstract

*Introduction*: Periacetabular osteotomy (PAO) is an effective joint-preserving procedure for young adults with developmental dysplasia of the hip. Although PAO provides excellent radiographic and clinical results, it is a technically demanding procedure with a distinct learning curve that requires careful 3D planning and, above all, has a number of potential complications. We therefore developed a pre-operative simulation method for PAO via creation of a new full-scale model.

*Methods*: The model was prepared from the patient’s Digital Imaging and Communications in Medicine (DICOM) formatted data from computed tomography (CT), for construction and assembly using 3D printing technology. A major feature of our model is that it is constructed from salt. In contrast to conventional models, our model provides a more accurate representation, at a lower manufacturing cost, and requires a shorter production time. Furthermore, our model realized simulated operation normally with using a chisel and drill without easy breakage or fissure. We were able to easily simulate the line of osteotomy and confirm acetabular version and coverage after moving to the osteotomized fragment. Additionally, this model allowed a dynamic assessment that avoided anterior impingement following the osteotomy.

*Results*: Our models clearly reflected the anatomical shape of the patient’s hip. Our models allowed for surgical simulation, making realistic use of the chisel and drill. Our method of pre-operative simulation for PAO allowed for the assessment of accurate osteotomy line, determination of the position of the osteotomized fragment, and prevented anterior impingement after the operation.

*Conclusion*: Our method of pre-operative simulation might improve the safety, accuracy, and results of PAO.

## Introduction

The Bernese periacetabular osteotomy (PAO) developed by Ganz et al. [1] is an effective joint-preserving procedure for young adults with developmental dysplasia of the hip (DDH). Curved periacetabular osteotomy (CPO), based on a modification of the Bernese PAO and developed by Naito et al. [2], involves the use of minimally invasive exposure and a spherical osteotomy to easily move the osteotomized fragment. While CPO can be utilized for early rehabilitation with fewer complications compared with the Bernese PAO, an outward curved osteotomy from the quadrilateral space is considered technically demanding, with surgeons having to perform the curved osteotomy outside of their field of vision.

Accurate assessment of acetabular morphology and its relationship to the femoral head is essential for pre-operative planning of PAO. However, while two-dimensional (2D) radiographs can be used to estimate acetabular coverage, only an experienced surgeon can successfully determine the osteotomy line and post-operative position of the osteotomized acetabular fragment. Here, we developed the procedure for pre-operative simulation for PAO via creation of a new full-scale model.

## Model and simulation technique

Rapid prototyping technique was used to quickly fabricate a scale model of each component, which was then assembled using three-dimensional (3D) computer-aided design (CAD) data. Components were then constructed and assembled using 3D printing technology, which has been widely used to test components at the manufacturing stage and recently used for full-scale models produced via rapid prototyping for surgical simulations [3]. Our model was prepared by extracting the patient’s Digital Imaging and Communications in Medicine (DICOM) format computed tomography (CT) data of the bone obtained by pre-operative scanning at an interval of 2 mm, converting it to 3D data and then to cross-sectional Windows bitmap (BMP) data at an interval of 0.2 mm. In contrast to conventional models, a major feature of our model is that it is constructed from salt. The conventional models have been constructed from plaster or acrylate resin. The grains of salt are much finer and cheaper than the materials used in a conventional model. The model was constructed via the repetitive application of specially developed ink contained adhesive to the material constructed from salt according to the cross-sectional data. Therefore, the model could provide a more accurate representation of bone structure (e.g. cortical and cancellous bone), at a lower manufacturing cost, and required a shorter production time. In addition, the current model was developed to be constructed in color (e.g. the vessels). The model allows surgeons to evaluate steps of the procedure not only pre-operatively but also intra-operatively, without any imaging equipment and any preparation intra-operatively. Furthermore, since specially developing the ink contained adhesive, our model allows simulated operation as real bone with using chisel and drill without easy breakage or fissure. In our model, the acetabular and femoral sides were created separately to allow a dynamic assessment. We first checked the morphology of the patient’s hip joint with respect to the morphology of the joint edge of the acetabulum and femoral head-neck junction as well as the width of the quadrilateral space. We then defined and measured the distance for three points considered to be along the osteotomy line as follows: from the osteotomy line of the iliac bone to the lateral joint edge, from the osteotomy line in the quadrilateral space to the accurate line and greater sciatic notch, and from the accurate line to the osteotomy line of the ischial bone. Additionally, we confirmed the direction and angle of application of the chisel for each part of the osteotomy to avoid intra-articular osteotomy. In addition, we measured the angle of application of the chisel and distance between osteotomy line and the specific point of the acetabulum (e.g. anterior inferior iliac spine, ischial spine, and iliopubic eminence). Finally, we cut the model along the osteotomy line and determined the position of the osteotomized fragment that would obtain enough coverage (i.e., a lateral center-edge [CE] angle ≥30°) to allow ≥90° of hip flexion with dynamic assessment without impingement between the femoral head-neck junction and acetabular rim. Additionally, we measured the angle and distance between the osteotomized fragment and host bone. Intra-operatively, we designed the osteotomy line according to the measurement during the pre-operative simulation and decided the position of the osteotomized fragment.

## Typical case presentation

**Figure 1.**
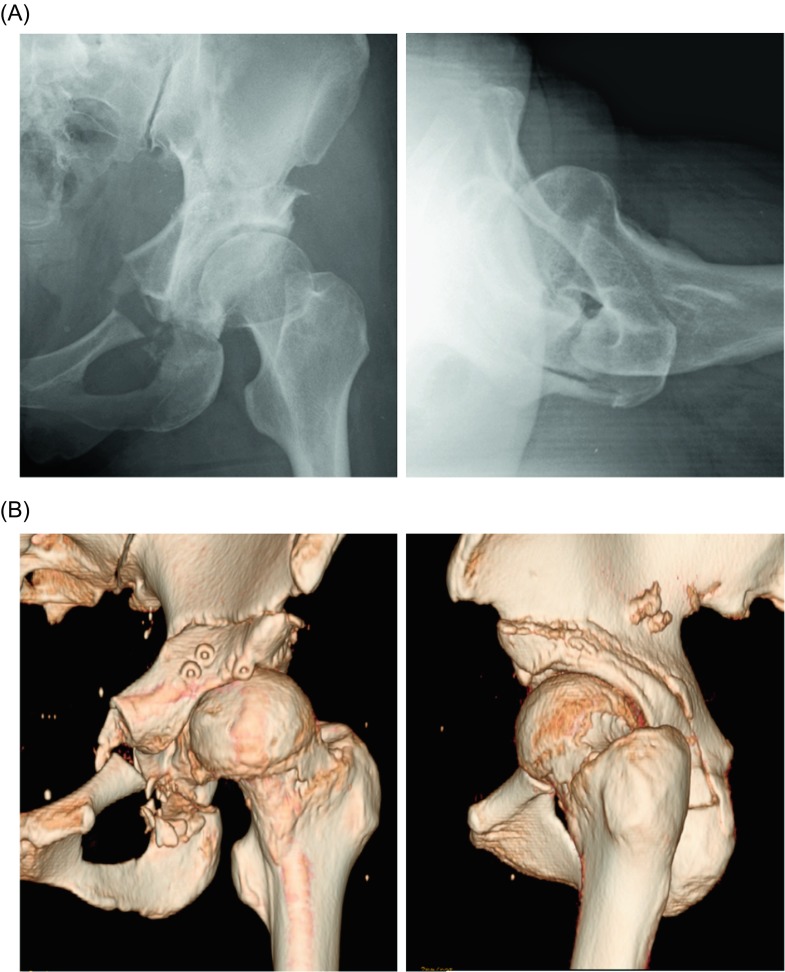
Pre-operative radiograph showing slight osteoarthritis due to hip dysplasia (A); bump on the femoral head-neck junction, with an alpha angle of 75° (B); pre-operative three-dimensional reconstruction of the computed tomography (CT) image (C).

A 37-year-old woman presented with pain in her left hip. A pre-operative radiograph revealed slight osteoarthritis due to hip dysplasia (Figure 1A). The lateral CE angle was 11°, and the femoral alpha angle was 75° (Figure 1B). Three-dimensional reconstruction via CT imaging revealed a bump on the femoral head-neck junction (Figure 1C).

**Figure 2.**
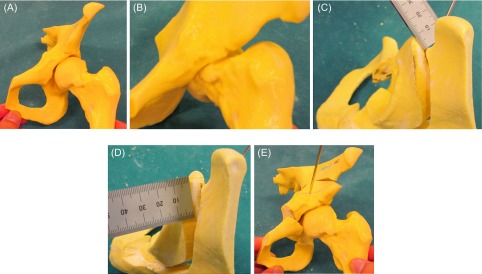
Three-dimensional salt-based model of hip (A); anterior impingement was simulated at general PAO position (B); the position of the fragment was 1 cm anterior (C) and lateral (D) from the original position was considered appropriate to avoid anterior impingement (E).

**Figure 3.**
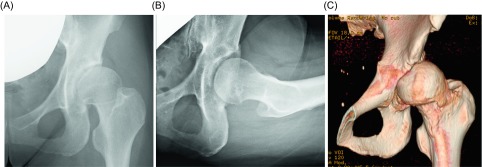
Post-operative radiograph following periacetabular osteotomy, which was aided by simulation with a three-dimensional (3D) salt-based model (A); post-operative 3D reconstruction computed tomography image clearly reflecting the pre-operative simulation (B).

We constructed a model for the patient for pre-operative simulation. The model clearly reflected the anatomical shape of the patient’s hip observed on imaging (Figure 2A). We could confirm easily anterior impingement when we moved the osteotomized fragment to the position routinely for PAO (i.e., 1 cm anteriorly and 1.5 cm laterally) (Figure 2B). Additionally, we estimated that anterior impingement would not be avoided if we added osteochondroplasty of the femoral neck. Therefore, we decided that the position of the fragment was 1 cm anterior (Figure 2C) and 1 cm lateral (Figure 2D) from the original position (Figure 2E) and added osteochondroplasty to avoid anterior impingement after the osteotomy. The post-operative radiograph showed efficient lateral coverage of the acetabulum (Figure 3A). Further, the lateral CE angle increased to 31°, and the 3D reconstruction CT showed that the osteochondroplasty had been performed accurately (Figure 3B). The original complaints of pain and limitation at the end of hip flexion were no longer present after the operation.

## Discussion

The DDH in adolescents and young adults is characterized by a shallow, obliquely oriented acetabulum. Anterior or lateral undercoverage with DDH results in significantly elevated contact pressures, reduced contact area, and instability; it remains one of the more frequent causes of secondary osteoarthritis of the hip [4]. As such, a number of acetabular reorientation operations have been indicated for DDH patients, with the Bernese PAO being the most widely used procedure for relieving pain and improving hip function [5]. Although the Bernese PAO provides excellent radiographic and clinical results, it is a technically demanding procedure with a distinct learning curve that requires careful 3D planning and, above all, has a number of potential complications [6]. The CPO technique developed by Naito et al. [2] uses a minimally invasive exposure to avoid extensive dissection of the abductor muscles from the outer aspect of the pelvis and a spherical osteotomy to avoid creating a gap between the host bone and osteotomized fragment. Thus, this procedure can be utilized for early rehabilitation, avoiding non-union of the ilium, and additional bone grafting. However, potential complications include intra-articular injuries, damage to the neurovascular structures, and posterior column discontinuity due to the limited visualization of the quadrilateral surface in the spherical osteotomy of the ilium.

The use of computer assistance in performing PAO has been described previously. Pre- and post-operative assessment of PAO using 3D-CT reconstruction provide exact anatomical information and facilitate a more accurate pre-operative representation than a 2D radiograph of femoral head coverage or other pre-operative simulations of the procedure [7, 8]. Langlotz et al. [9] and Hsieh et al. [10] described intra-operative CT-based navigation guidance for the Bernase periacetabular osteotomy and PAO, respectively. Akiyama et al. [11] also reported cases of CPO operation performed under intra-operative CT-based navigation guidance. All of these studies reported that a CT-based navigation system aided visualization and significantly contributed to increased accuracy and safety. However, reported disadvantages of this method include considerable cost to acquire the navigation system and the time for registration during surgery, which could prolong surgery and increase the risk of hemorrhage and infection. In the current technique, we only have to prepare the patient’s CT data. The patient had no additional exposure to radiation, as routine CT scans are conducted. Including the cost of processing the original CT image, the manufacturing cost was $500 per model.

Regarding another limitation of CT-based navigation, namely that the movement and position of the osteotomized fragment are not tracked or measured [11], the present method could be used to assess and experiment with the appropriate position of the osteotomized fragment to avoid anterior impingement. Anterior impingement after PAO, which occurs at a high rate (47.8%) in male patients [12], can lead to unsatisfactory clinical results and progression of osteoarthritis. Although predicting anterior impingement after PAO is currently difficult, the optimum position of the osteotomized fragment to avoid anterior impingement can be decided pre-operatively. Since our method can be utilized to simulate motion pre-operatively, analyzing the impingement between the osteotomized fragment and femoral bone can be performed easily and correctly, ultimately avoiding anterior impingement after PAO. Consequently, our method might improve the safety, accuracy, and results for PAO surgery.

The major limitation of current study warrants mention. Since this method has been indicated for small number of cases so far, we could not investigate objective assessment of reproductively of our methods in the current study. Further study is needed to determine its efficacy.

## Conflict of interest

All authors declare that no benefits in any form that are related directly or indirectly to the subject of this manuscript have been or will be received from a commercial party.
